# End-of-season influenza vaccine effectiveness in adults and children, United Kingdom, 2016/17

**DOI:** 10.2807/1560-7917.ES.2017.22.44.17-00306

**Published:** 2017-11-02

**Authors:** Richard Pebody, Fiona Warburton, Joanna Ellis, Nick Andrews, Alison Potts, Simon Cottrell, Arlene Reynolds, Rory Gunson, Catherine Thompson, Monica Galiano, Chris Robertson, Naomh Gallagher, Mary Sinnathamby, Ivelina Yonova, Ana Correa, Catherine Moore, Muhammad Sartaj, Simon de Lusignan, Jim McMenamin, Maria Zambon

**Affiliations:** 1 Public Health England, London, United Kingdom; 2 Health Protection Scotland, Glasgow, United Kingdom; 3 Public Health Wales, Cardiff, United Kingdom; 4 West of Scotland Specialist Virology Centre, Glasgow, United Kingdom; 5 University of Strathclyde, Glasgow, United Kingdom; 6 Public Health Agency Northern Ireland, Belfast, United Kingdom; 7 University of Surrey, Guildford, United Kingdom; 8 Royal College of General Practitioners, London, United Kingdom

**Keywords:** Influenza, Influenza virus, influenza-like illness - ILI, vaccines and immunization, vaccine effectiveness, LAIV

## Abstract

The United Kingdom is in the fourth season of introducing a universal childhood influenza vaccine programme. The 2016/17 season saw early influenza A(H3N2) virus circulation with care home outbreaks and increased excess mortality particularly in those 65 years or older. Virus characterisation data indicated emergence of genetic clusters within the A(H3N2) 3C.2a group which the 2016/17 vaccine strain belonged to. **Methods: **The test-negative case–control (TNCC) design was used to estimate vaccine effectiveness (VE) against laboratory confirmed influenza in primary care. **Results: **Adjusted end-of-season vaccine effectiveness (aVE) estimates were 39.8% (95% confidence interval (CI): 23.1 to 52.8) against all influenza and 40.6% (95% CI: 19.0 to 56.3) in 18–64-year-olds, but no significant aVE in ≥ 65-year-olds. aVE was 65.8% (95% CI: 30.3 to 83.2) for 2–17-year-olds receiving quadrivalent live attenuated influenza vaccine. **Discussion: **The findings continue to provide support for the ongoing roll-out of the paediatric vaccine programme, with a need for ongoing evaluation. The importance of effective interventions to protect the ≥ 65-year-olds remains.

## Introduction

The United Kingdom (UK) has a long-standing influenza selective immunisation programme offering inactivated vaccine to people 65 years of age and older and those 6 months to less than 65 years of age with an underlying clinical risk factor [1]. Following advice from the Joint Committee on Vaccination and Immunisation (JCVI), the UK started the incremental introduction of a universal childhood influenza vaccine programme in the 2013/14 influenza season [2] with a newly licensed intra-nasally administered live attenuated influenza vaccine (LAIV). Eligible healthy children were offered a single dose of LAIV, whereas children in a clinical risk group up to 9 years of age, with no contraindications for LAIV and not previously vaccinated, were offered two doses of vaccine. By the 2016/17 season, all children aged 2–8 years across the UK were being offered quadrivalent LAIV (LAIV4), or else quadrivalent inactivated vaccine (QIV) if LAIV4 was contraindicated [1]. In addition, Scotland and Northern Ireland offered LAIV4 also to all remaining children of primary school age up to 11 years of age. The UK has found evidence of LAIV4 effectiveness in 2015/16 of 58% and continues to recommend its use [3]. This is in contrast to the United States (US) where there has been a longstanding paediatric influenza vaccination programme, and where reduced LAIV vaccine effectiveness (VE) has been described by the US Centres for Disease Control (CDC). This led to a recommendation to suspend use of LAIV in children in 2016/17 [4] and important questions about what might explain these observations of apparent reduced LAIV effectiveness, including what role prior vaccination may play [5].

The 2016/17 influenza season in the UK, as with many other northern hemisphere countries, was characterised by the early circulation of influenza A(H3N2) viruses. The season started in December 2016 and peaked over the Christmas/New Year period. It was characterised by large numbers of care home outbreaks, many of which included highly vaccinated populations, increased admissions to hospital compared with the previous season and significant excess mortality particularly among those 65-year-old or older, despite vaccine uptake levels of over 70% in this age group [6]. Questions have been previously raised about the effectiveness of inactivated influenza vaccine in older persons and a range of potential explanatory factors have been postulated including what role prior vaccine exposure may play in reducing VE in this age group [7].

The UK has a well-established system to monitor influenza VE each season, including mid-season estimates based upon sentinel swabbing in primary care [3,8]. Here we present the 2016/17 end-of-season VE findings for laboratory-confirmed infection in primary care across all age groups, with a particular focus on LAIV4 in children and inactivated influenza vaccine (IIV) in adult age groups and we explore the possible effect of prior season vaccination on VE in the current season. A comparison with the mid-season estimate is also undertaken.

## Methods

The test-negative case–control (TNCC) design was used to estimate VE, with the study undertaken in the registered population of five sentinel general practice (GP) surveillance networks across the UK. All undertake respiratory swabbing, with details of these schemes outlined previously [3]. The schemes are: the Royal College of General Practitioners (RCGP) Research and Surveillance Centre (RSC) network, the Public Health England (PHE) Specialist Microbiology Network (SMN) and the national sentinel schemes of Wales, Scotland and Northern Ireland.

The study took place in the period from 1 October 2016, the time when influenza surveillance in primary care with respiratory swabbing was started, until 19 March 2017. A mid-season analysis was undertaken for samples taken up to 15 January 2017, in order to provide results to the annual World Health Organization (WHO) meeting on the composition of influenza virus vaccines for the next influenza season [9]. The study population comprised patients presenting to their GP during the study period with an acute influenza-like illness (ILI), who the physician consented verbally and swabbed during the consultation. The majority of the UK influenza vaccine programme is delivered in the period late September through to the end November [6].

### Definition of cases and controls

A case of ILI was defined as an individual who presented with an acute respiratory illness with physician-diagnosed fever or complaint of feverishness in the previous 7 days. Participating GPs were asked to invite persons presenting with ILI to provide a swab for diagnosis. Swabbing was undertaken regardless of vaccination status. Cases were patients who tested positive for seasonal influenza A or B virus by real-time PCR testing. Controls were patients with the same symptoms who tested negative for influenza A or B virus.

### Data collection

During the consultation, the GP completed a standard questionnaire. This collected demographic (age and sex), clinical (date of onset and history of fever) and epidemiological information from patients including vaccination status and potential confounders such as underlying clinical risk factors. Vaccination status including date of vaccination was obtained mainly from patient records. Vaccine type (LAIV4 intranasal; QIV injectable) was specified on the form. Time since vaccination was stratified into <3 months and >3 months until onset of illness. Study subjects were categorised according to Department of Health defined risk categories for influenza vaccination [1]. High risk was determined by the presence of well recognised risk morbidities recorded in the electronic health record for the patient concerned. In addition, it was noted whether the general practice was in a pilot area for England-based paediatric immunisation schemes, where all primary school age children were offered LAIV4.

Patients were defined as vaccinated if they were reported to have received the 2016/17 seasonal vaccine at least 14 days before first onset of symptoms. Patients were excluded if they were vaccinated less than 14 days before symptom onset. If date of vaccination was unknown, it was assumed to be 15 October 2016, which was the median of all known vaccination dates this season (data not shown).

Registered patients were excluded if they had expressed a wish to be, or the practice used one of the codes that they may not want to share data (e.g. Summary Care Record or Care.data opt-out codes).

### Laboratory methods

GPs took combined throat and nose swabs which were then sent from the practice to the usual laboratory. Samples in England were sent to the PHE Reference Virus Unit, Colindale (RCGP scheme) or one of the specialist regional microbiology laboratories (SMN scheme). Samples in Wales were sent to the Public Health Wales Specialist Virology Centre and in Scotland to the West of Scotland Specialist Virology Centre (HPS scheme) (WoSPV). In Northern Ireland samples were sent to the Regional Virus Laboratory, Belfast. Influenza laboratory confirmation was made using comparable real-time PCR methods able to detect circulating influenza A and influenza B viruses [10]. All laboratories then sent influenza virus-positive detections to the reference laboratories for further characterisation (RVU in London for all schemes except in Scotland where they were sent to WoSPV in Glasgow). Isolation of influenza viruses was attempted from all suitable PCR positive samples using Madin-Darby canine kidney epithelial (MDCK) cells or MDCK cells containing the cDNA of human 2,6-sialtransferase (SIAT1) cells [11,12].

Virus isolates with a haemagglutination titre ≥ 40 were characterised antigenically using post-infection ferret antisera in haemagglutination inhibition (HI) assays, with guinea pig (A(H3N2) viruses) or turkey (influenza B viruses) red blood cells [12]. Reference virus strains used for HI assays included A/HongKong/4801/2014, B/Brisbane/60/2008, B/Phuket/3073/2013 (vaccine strains) and other A(H3N2) and influenza B reference strains grown in embryonated chicken eggs and tissue culture cells.

Nucleotide sequencing of the haemagglutinin (HA) gene of a subset of influenza A(H3N2) and influenza B viruses selected to be representative of the range of the patients’ age, date of sample collection, geographical location and antigenic characterisation of the virus isolate, if performed, was undertaken. Phylogenetic trees of the HA gene of A(H3N2) viruses were constructed with a neighbour-joining algorithm available in the Mega 6 software (http://www.megasoftware.net) [13].

HA sequences from reference strains used in the phylogenetic analysis were obtained from the EpiFlu database of the Global Initiative on Sharing Avian Influenza Data (GISAID) (Table 1). The HA sequences generated for this study and used in the phylogenetic analysis, were deposited in GISAID’s EpiFlu database under the following accession numbers: EPI913897, EPI913905, EPI913913, EPI913922, EPI913930, EPI913938, EPI913946, EPI913954, EPI913962, EPI913970, EPI913978, EPI913986, EPI913994, EPI914010, EPI914018, EPI914026, EPI914034, EPI914042, EPI914050, EPI914058, EPI914066, EPI914074, EPI914082, EPI914090, EPI914098, EPI914106, EPI914114, EPI914122, EPI914130, EPI914138, EPI914146, EPI914154, EPI914162, EPI914170, EPI914178, EPI914186, EPI914194, EPI914202, EPI914210, EPI914218, EPI914226, EPI914234, EPI914242, EPI914250, EPI914258, EPI914266, EPI914274, EPI914282, EPI914290, EPI914314, EPI914322, EPI914330, EPI914338, EPI914346, EPI914354, EPI914362, EPI914370, EPI914378, EPI914386, EPI914402, EPI914410, EPI914426, EPI914434, EPI914442, EPI914450, EPI914458, EPI914466, EPI914474, EPI914482, EPI914490, EPI914498, EPI914506, EPI914763, EPI914771, EPI914779, EPI914787, EPI914795.

**Table 1:** Details of influenza A(H3N2) haemagglutinin sequences obtained from GISAID used in the phylogenetic analysis, test–negative case–control study, United Kingdom, 2016/17 influenza season

Virus isolate	Segment ID/Accession number	Country	Collection date	Originating laboratory	Submitting laboratory
A/Samara/73/2013	EPI460558	Russian Federation	12 Mar 2013	WHO National Influenza Centre, Saint Petersburg, Russian Federation	National Institute for Medical Research, London, UK
A/Switzerland/9715293/2013	EPI530687	Switzerland	6 Dec 2013	Hopital Cantonal Universitaire de Geneves, Switzerland	National Institute for Medical Research, London, UK
A/Hong Kong/4801/2014	EPI539576	Hong Kong (SAR)	26 Feb 2014	Government Virus Unit, Hong Kong (SAR)	National Institute for Medical Research, London, UK
A/New Caledonia/71/2014	EPI551570	New Caledonia	13 Aug 2014	Institut Pasteur New Caledonia, New Caledonia	WHO Collaborating Centre for Reference and Research on Influenza, Melbourne, Australia
A/Texas/50/2012	EPI556816	United States	15 Apr 2012	Texas Department of State Health Services-Laboratory Services, Austin, US	Centers for Disease Control and Prevention, Atlanta, US
A/England/525/2014	EPI611375	UK	20 Nov 2014	Microbiology Services Colindale, Public Health England, London, UK	Microbiology Services Colindale, Public Health England, London, UK
A/England/507/2014	EPI626573	UK	24 Aug 2014	Microbiology Services Colindale, Public Health England, London, UK	Microbiology Services Colindale, Public Health England, London, UK
A/Bolzano/7/2016	EPI773595	Italy	15 Mar 2016	Istituto Superiore di Sanità, Rome, Italy	Crick Worldwide Influenza Centre, London, UK
A/Scotland/63440583/2016	EPI831436	UK	25 Aug 2016	Gart Naval General Hospital, Glasgow, Scotland, UK	Microbiology Services Colindale, Public Health England, London, UK

GISAID: Global Initiative on Sharing All Influenza Data; SAR: Special Administrative Region of the People's Republic of China; UK: United Kingdom; US: United States; WHO: World Health Organization.

### Statistical methods

To analyse the swabbing results (test–negative design), the odds ratio (OR) of being vaccinated between cases and controls was used to calculate the crude VE as (1–OR) x 100%. We performed a multivariable logistic regression, as previously [1,8], to adjust VE for potential confounders with influenza laboratory results as the outcome and influenza vaccination status as the linear predictor. Estimates were calculated adjusting for age (by < 2, 2–11, 12–17, 18–44, 45–64 and ≥ 65 years), month of onset of symptoms, surveillance scheme, sex, and residence in area where a primary school programme was in place. The effect of adjustment for risk group was also assessed. Stratification was by age 2–17, 18–64 and ≥ 65 years and was split by vaccine type (LAIV/QIV) within those aged 2–17 years. The effect of prior season vaccination was also described by calculating all the VEs i.e. for vaccinated in both 2016/17 and 2015/16 seasons, vaccinated only in the 2015/16 season and vaccinated only in the 2016/17 season and by comparing to not vaccinated in either season. VE was calculated as 1-OR, where OR is the odds of vaccination in cases compared with controls.

End-of-season results were compared to mid-season estimates to determine the accuracy of the latter estimates.

The collection and analysis of swab forms according to positivity was undertaken as part of routine influenza surveillance, with the swabs taken to assist clinical management. The collection of the clinical data accords with routine usual practice in public health. Specific ethical approval was not necessary.

## Results

**Figure 1:**
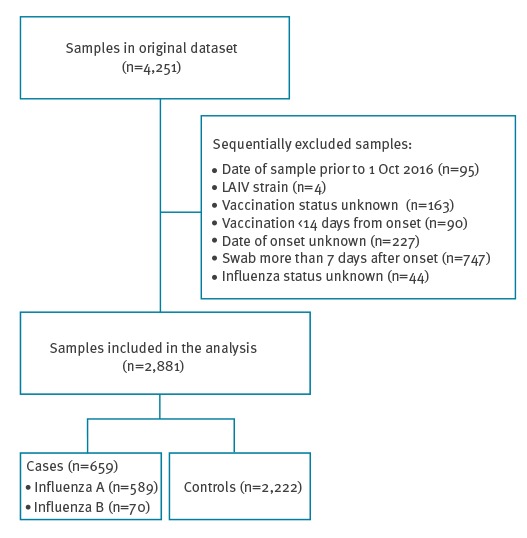
Swabbing results of patients with influenza-like illness in primary care during the influenza season, United Kingdom, October 2016–March 2017

During the study, 4,251 persons were sampled in the participating sentinel primary care practices and were tested. The reasons for study exclusion are summarised in Figure 1.

LAIV: live attenuated influenza vaccine.

The details of the 2,881 individuals remaining in the study were stratified according to the swab result (Table 2) and by vaccine status (Table 3). There were 2,222 controls and 659 cases, of whom 589 were influenza A (H3N2 n=514; A unknown n=70; H1N1pdm09 n=5) and 70 were influenza B. The first positive influenza virus detection was made on 14 October 2016.

**Table 2:** Details for influenza A and B cases and controls, test–negative influenza case–control study, United Kingdom, October 2016–March 2017 (n=659 cases and 2,222 controls)

Characteristic	Controls (n = 2,222)		Influenza B (n = 70)		Influenza A(H3N2) (n = 514)		Influenza A(H1N1)pdm09 (n = 5)		Influenza A unknown (n = 70)		p value
	n	%	n	%	n	%	n	%	n	%	
**Age (years)**											
< 2	125	87.4	1	0.7	15	10.5	0	0	2	1.4	< 0.0001
2–11	313	83.5	5	1.3	52	13.9	1	0.3	4	1.1	
12–17	146	69.9	9	4.3	50	23.9	0	0	4	1.9	
18–44	774	76.1	26	2.6	187	18.4	1	0.1	29	2.9	
45–64	534	74.3	17	2.4	140	19.5	2	0.3	26	3.6	
≥65	313	78.6	12	3	67	16.8	1	0.3	5	1.3	
Missing information	17	85	0	0	3	15	0	0	0	0	
**Sex**											
Female	1,352	78.1	45	2.6	293	16.9	3	0.2	39	2.3	0.144
Male	864	75.7	25	2.2	219	19.2	2	0.2	31	2.7	
Missing	6	75	0	0	2	25	0	0	0	0	
**Surveillance scheme**											
NI	75	66.4	4	3.5	30	26.5	2	1.8	2	1.8	< 0.0001
RCGP	720	74.2	0	0	250	25.7	1	0.1	0	0	
SMN	107	74.3	2	1.4	34	23.6	0	0	1	0.7	
Scotland	1,233	81.7	62	4.1	147	9.7	1	0.1	67	4.4	
Wales	87	60.8	2	1.4	53	37.1	1	0.7	0	0	
**Risk group**											
No	1,379	76.2	46	2.5	335	18.5	3	0.2	46	2.5	0.106
Yes	654	79.1	18	2.2	136	16.4	2	0.2	17	2.1	
Missing	189	77.1	6	2.4	43	17.6	0	0	7	2.9	
**Symptom onset to swab (days)**											
0–1	262	75.1	4	1.1	76	21.8	1	0.3	6	1.7	< 0.0001
2–4	1,165	74.9	45	2.9	300	19.3	2	0.1	44	2.8	
5–7	795	81.5	21	2.2	138	14.1	2	0.2	20	2	
**Vaccination status**											
Unvaccinated	1,642	76.4	55	2.6	389	18.1	5	0.2	57	2.7	0.016
Vaccinated (14–91 days ago)	347	82.4	5	1.2	61	14.5	0	0	8	1.9	
Vaccinated (> 91 days ago)	233	74.7	10	3.2	64	20.5	0	0	5	1.6	
**Prior vaccination seasons^a^ (age group 2–17-year-olds LAIV only)**											
Unvaccinated 2016/17 and 2015/16	240	74.5	10	3.1	65	20.2	1	0.3	6	1.9	< 0.0001
Vaccinated 2015/16 only	61	91	2	3	4	6	0	0	0	0	
Vaccinated 2016/17 only	27	75	1	2.8	8	22.2	0	0	0	0	
Vaccinated 2015/16 and 2016/17	63	95.5	0	0	3	4.6	0	0	0	0	
**Prior vaccination seasons^a^ (age group 18 years and older only)**											
Unvaccinated 2016/17 and 2015/16	837	75.5	26	2.3	208	18.8	1	0.1	37	3.3	< 0.0001
Vaccinated 2015/16 only	142	90.4	2	1.3	11	7	0	0	2	1.3	
Vaccinated 2016/17 only	50	76.9	4	6.2	8	12.3	0	0	3	4.6	
Vaccinated 2015/16 and 2016/17	351	79.2	5	1.1	79	17.8	0	0	8	1.8	
**Pilot area (RCGP and SMN only)**											
No	804	73.8	2	0.2	281	25.8	1	0.1	1	0.1	0.092
Yes	23	88.5	0	0	3	11.5	0	0	0	0	
**Month of onset of illness**											
October	419	99.3	0	0	3	0.7	0	0	0	0	< 0.0001
November	486	94.9	2	0.4	20	3.9	1	0.2	3	0.6	
December	493	72.9	5	0.7	170	25.1	1	0.1	7	1	
January	429	58.8	24	3.3	234	32.1	3	0.4	39	5.3	
February	271	68.8	27	6.9	78	19.8	0	0	18	4.6	
March	124	83.8	12	8.1	9	6.1	0	0	3	2	
**Vaccine type (2–17-year-olds only)**											
Not Vaccinated	344	75.8	13	2.9	89	19.6	1	0.2	7	1.5	NA
Injection	9	81.8	0	0	2	18.2	0	0	0	0	
Intranasal/LAIV	101	89.4	1	0.9	11	9.7	0	0	0	0	
Missing information	5	83.3	0	0	0	0	0	0	1	16.7	

LAIV: live attenuated influenza vaccine; NA: not applicable; NI: Northern Ireland; PHE: Public Health England; RCGP: Royal College of General Practitioners’ Research and Surveillance Centre (RSC) scheme; SMN: PHE Specialist Microbiology Network.

Numbers and row percentages to indicate positivity rates^a^ are shown.

^a^ Only includes those whose prior vaccination status was known.

**Table 3:** Key demographic variables by influenza vaccination status, test–negative influenza case–control study, United Kingdom, October 2016–March 2017 (n=659 cases and 2,222 controls)

Characteristic	Not vaccinated (n = 2,148)		Vaccinated (n = 733)		p value
	n	%	n	%	
**Age group (years)**					
< 2	143	100	0	0	< 0.0001
2–11	262	69.9	113	30.1	
12–17	192	99.5	1	0.5	
18–44	884	86.9	133	13.1	
45–64	521	72.5	198	27.5	
≥65	131	32.9	267	67.1	
Missing	15	75	5	25	
**Sex**					
Female	1,274	73.6	458	26.4	0.144
Male	867	76	274	24	
Missing information	7	87.5	1	12.5	
**Surveillance scheme**					
NI	84	74.3	29	25.7	0.675
RCGP	735	75.7	236	24.3	
SMN	110	76.4	34	23.6	
Scotland	1,109	73.4	401	26.6	
Wales	110	76.9	33	23.1	
**Risk group**					
No	1,597	88.3	212	11.7	< 0.0001
Yes	366	44.3	461	55.7	
Missing information	185	75.5	60	24.5	
**Onset to swab (days)**					
0–1	271	77.7	78	22.3	0.305
2–4	1,147	73.7	409	26.3	
5–7	730	74.8	246	25.2	
**Pilot area (RCGP and SMN only)**					
No	824	75.7	265	24.3	0.548
Yes	21	80.8	5	19.2	
**Month of onset of illness**					
October	388	91.9	34	8.1	< 0.0001
November	393	76.8	119	23.2	
December	469	69.4	207	30.6	
January	510	70	219	30	
February	276	70.1	118	29.9	
March	112	75.7	36	24.3	

NI: Northern Ireland; RCGP: Royal College of General Practitioners’ Research and Surveillance Centre (RSC) scheme; SMN: PHE Specialist Microbiology Network

Numbers and row percentages (to indicate vaccination rates) are shown.

### Virus characterisation

**Figure 2:**
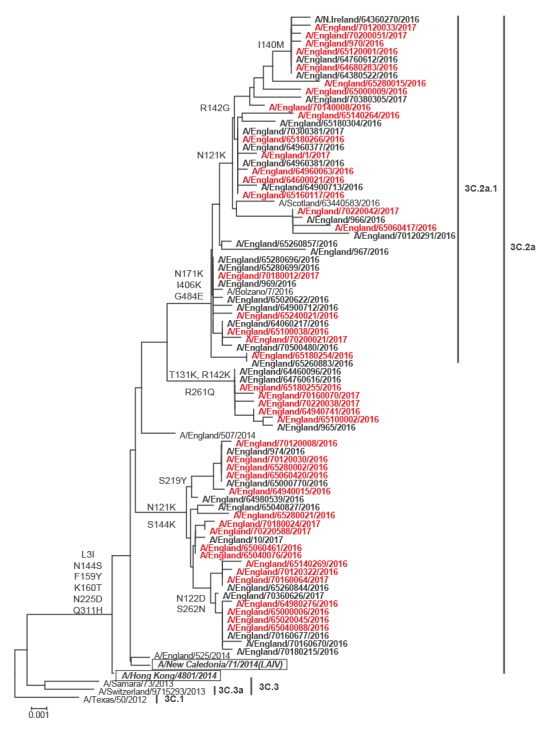
Phylogenetic analysis of full length haemagglutinin gene comparing reference sequences from the GISAID EpiFlu database and influenza A(H3N2) sequences from patients, United Kingdom, influenza season 2016/17

GISAID: Global Initiative on Sharing Avian Influenza Data; UK: United Kingdom.

Figure 2 shows the phylogenetic analysis of the HA sequences for A(H3N2) in the 2016/17 season. Genetic characterisation of 416 A(H3N2) influenza viruses from all sources since week 40 showed that they all belonged to genetic subclade 3C.2a, with 220 (52.9%) belonging to a cluster within this genetic subclade designated as 3C.2a1. The northern hemisphere 2016/17 influenza A(H3N2) vaccine strain A/HongKong/4801/2014 belongs to genetic subclade 3C.2a and its relatedness to the circulating strains in the 2016/17 season is shown in Figure 2. The common signature amino-acid substitutions characterising genetic groups in 3C.2a viruses are shown at the root of each cluster on the tree. The 2016/17 season’s A(H3N2) viruses were difficult to cultivate, and only 23 influenza A(H3N2) viruses were antigenically characterised since week 40 2016, representing a minority of the detections and thus a potential bias in the available antigenic data. The viruses antigenically analysed were similar to the A/HongKong/4801/2014 northern hemisphere 2016/17 A(H3N2) vaccine strain. Of the 23 antigenically characterised viruses, eight isolates were also genetically characterised, with all belonging to genetic group 3C.2a, and six of them belonging to the recently emerged 3C.2a1 cluster.

The tree was built using a neighbour-joining algorithm, with vaccine strain A/Texas/50/2012 selected as the root. Signature amino acid substitutions characterising genetic groups are annotated at the root of each cluster. UK 2016/17 samples are highlighted in bold. Sentinel samples are highlighted in red.

Genetic characterisation of 62 influenza B viruses from all sources was completed, with 58 (93.5%) viruses classified as belonging to the B/Yamagata/16/88, and genetically similar to B/Phuket/3073/2013, the influenza B/Yamagata/16/88 component of the 2016/17 northern hemisphere quadrivalent vaccine, and four (6.5%) classified as falling in the B/Victoria/2/87-lineage and genetically similar to B/Brisbane/60/2008, the influenza B/Victoria/2/87 lineage component of 2016/17 northern hemisphere trivalent and quadrivalent vaccines.

Eighteen influenza B viruses were isolated and antigenically characterised since week 40 2016; 13 viruses were characterised as belonging to the B/Yamagata/16/88-lineage and antigenically similar to B/Phuket/3073/2013, the influenza B/Yamagata-lineage component of 2016/17 northern hemisphere quadrivalent vaccine, and five (27.8%) viruses were characterised as belonging to the B/Victoria/2/87-lineage and antigenically similar to B/Brisbane/60/2008, the influenza B/Victoria-lineage component of 2016/17 northern hemisphere trivalent and quadrivalent vaccines.

### Model fitting for vaccine effectiveness estimation

When estimating VE, age group, sex, time period (defined by month of sample collection), surveillance scheme and primary school age pilot programme area were adjusted for in a multivariable logistic regression model. All variables that were adjusted for, except for sex and primary school age pilot programme in England, were significantly associated with a positive swab (Table 2). Only month was a confounder for the vaccine effects (changing the overall estimate by more than 5%). For any influenza (A and B), the crude VE was 14.4% (95% CI: -5.0% to 30.2%), which increased to 38.2% (23.4% to 50.2%), when month was included in the model. The crude and adjusted VE (aVE) estimates against all influenza, influenza A(H3N2) and B are shown in Table 4. The aVE point estimate of influenza vaccine against any laboratory-confirmed infection was 39.8% (95% CI: 23.1 to 52.8) and was similar for influenza A(H3N2) at 31.6% (95% CI: 10.3 to 47.8), reflecting the fact that A(H3N2) was the dominant circulating strain in the season. There were inadequate numbers of detections to enable estimation of effectiveness against influenza A(H1N1)pdm09.

**Table 4:** Influenza cases and controls according to vaccination status and VE estimates, test–negative influenza case–control study, United Kingdom, October 2016–March 2017 (n=659 cases and 2,222 controls)

Influenza type	Cases		Controls		Crude VE (95% CI)	Adjusted^a^ VE (95% CI)
	Vaccinated	Unvaccinated	Vaccinated	Unvaccinated		
** A or B**	153	506	580	1,642	14.4 (-5.0 to 30.2)	39.8 (23.1 to 52.8)
** A**	138	451	580	1,642	13.4 (-7.2 to 30.0)	36.7 (18.3 to 51.0)
** A(H3N2)**	125	389	580	1,642	9.0 (-13.7 to27.2)	31.6 (10.3 to 47.8)
** B**	15	55	580	1,642	22.8 (-37.7 to 56.7)	54.5 (10.8 to 76.8)

CI: confidence interval; VE: vaccine effectiveness.

^a^ Adjusted for age group, sex, month, pilot area and surveillance scheme.

A secondary analysis was undertaken including risk factor in the model. This led to only small changes (< 5%) in the point estimates, but wider confidence intervals due to loss of data, with an overall aVE point estimate for all influenza of 36.8% (95% CI: 16.9 to 52.0). Further sensitivity analyses were undertaken. Firstly, including all swabs no matter how long after onset they had been taken, which made less than 3% difference to the overall VE point estimate. Then a model including those vaccinated within 14 days as unvaccinated and including all swabs regardless of time since onset of symptoms. There was again < 3% difference to the VE point estimate.

Genetic characterisation information was available for 153 influenza A(H3N2) detections, with 66 3C.2a and 87 3C.2a1 detections giving an all age adjusted VE point estimate against 3C.2a1 of 22.7% (95% CI: -35.3 to 55.9) and for 3C.2a of 43.4% (95% CI: -12.3 to 71.5). The adjusted VE point estimate against influenza B was 54.5% (95% CI: 10.8 to 76.8). There were 42 B/Yamagata/16/88-lineage study samples with available genetic information giving an adjusted VE of 58.5% (95% CI: 3.1 to 82.2).

### Vaccine effectiveness against influenza A(H3N2)

#### In adults

Table 5 shows the age-specific crude and aVE by sub-type. The VE point estimate for inactivated vaccine (IIV) in 18–64-year-olds for influenza A(H3N2) was 36.6% (95% CI: 10.4 to 55.1), however, there was no significant effectiveness against influenza A or specifically A(H3N2) in those aged 65 years and above (aVE: -68.4%; 95% CI: -248.9 to 18.7). Although further analysis found no statistically significant decline in effectiveness by age in ≥ 18-year-olds (p = 0.516 for linear trend on log-odds), the direction of the trend was towards a decrease in VE with age and this was more rapid in those aged ≥ 65 years.

**Table 5:** Adjusted vaccine effectiveness estimates for influenza by sub-type, age group and vaccine type, test–negative case–control study, United Kingdom, October 2016–March 2017 (n=659 cases and 2,222 controls)

Influenza type/subtype and age group	Cases		Controls		Crude VE (95% CI)	Adjusted^a^ VE (95% CI)
**A and B**	**Vaccinated**	**Unvaccinated**	**Vaccinated**	**Unvaccinated**		
2–17 (IIV)	2	110	9	344	30.5 (-226.5 to 88.6)	43.2 (-183.5 to 88.6)
2–17 (LAIV4)	12	110	101	344	62.8 (29.8 to 80.3)	65.8 (30.3 to 83.2)
18–64	75	353	256	1,052	12.7 (-16.0 to 34.3)	40.6 (19.0 to 56.3)
≥ 65	63	22	204	109	-53 (-162.1 to 10.7)	-6.3 (-94.5 to 42)
**A**	**Vaccinated**	**Unvaccinated**	**Vaccinated**	**Unvaccinated**		
2–17 (IIV)	2	97	9	344	21.2 (-270.8 to 83.3)	30.9 (-260.3 to 86.7)
2–17 (LAIV4)	11	97	101	344	61.4 (25.1 to 80.1)	63.3 (22.0 to 82.7)
18–64	69	316	256	1,052	10.3 (-20.4 to 33.1)	38.5 (15.1 to 55.3)
≥ 65	55	18	204	109	-63.3 (-191.8 to 8.7)	-21.2 (-134.4 to 37.3)
**A(H3N2)**	**Vaccinated**	**Unvaccinated**	**Vaccinated**	**Unvaccinated**		
2–17 (IIV)	2	89	9	344	14.1 (-304.6 to 81.8)	24.9 (-296.1 to 85.8)
2–17 (LAIV4)	11	89	101	344	57.9 (18.2 to 78.3)	57 (7.7 to 80.0)
18–64	59	268	256	1,052	9.5 (-23.7 to 33.9)	36.6 (10.4 to 55.1)
≥ 65	53	14	204	109	-102.3 (-281.0 to -7.4)	-68.4 (-248.9 to 18.7)
**B**	**Vaccinated**	**Unvaccinated**	**Vaccinated**	**Unvaccinated**		
2–17 (LAIV4)	1	13	101	344	73.8 (-102.7 to 96.6)	78.6 (-86.0 to 97.5)
18–64	6	37	256	1,052	33.4 (-59.6 to 72.2)	52.1 (-20.0 to 80.9)
≥ 65	8	4	204	109	-6.9 (-262.9 to 68.5)	17.2 (-249.7 to 80.4)

CI: confidence interval; IIV: inactivated influenza vaccine; LAIV4: quadrivalent live attenuated influenza vaccine; VE: vaccine effectiveness.

**^a^** Adjusted for age group, sex, month, pilot area and surveillance scheme.

**Figure 3:**
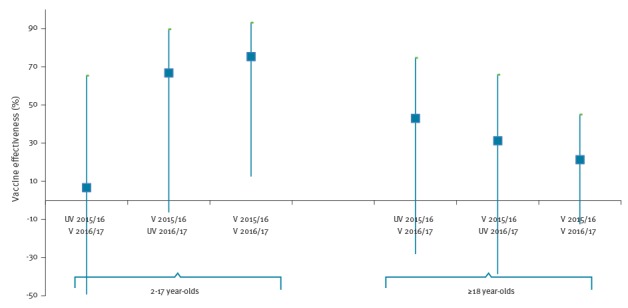
Adjusted^a^ vaccine effectiveness estimates for influenza A(H3N2) by prior vaccination status and sub-type in children 2–17 years of age^b^ and adults ≥ 18 years, test–negative case–control study, United Kingdom, October 2016–March 2017 (n=659 cases and 2,222 controls)

UV: unvaccinated; V: vaccinated.

^a^ Adjusted for age group, sex, month, pilot area and surveillance scheme.

^b^ Live attenuated influenza vaccine only.

In relation to vaccination in the prior season in the ≥18-year-olds (Figure 3), the VE point estimate was lowest in those vaccinated in both 2016/17 and 2015/16 seasons (21.3%; 95% CI: -12.5 to 44.9) and higher in those vaccinated only in the 2016/17 season (42.9%; 95% CI: -28.1 to 74.6), although the results were not statistically significant.

#### In children

The crude and aVE in children 2–17 years of age for LAIV4 and IIV is shown in Table 5. The aVE point estimate for LAIV4 against influenza A(H3N2) in children 2–17 years old was 57% (95% CI: 7.7 to 80), and non-significantly lower for IIV at 24.9% (95% CI: -296.1 to 85.8).

Figure 3 shows the influence of LAIV4 vaccination in 2–17-year-olds in the prior season, with the VE point estimate highest in those vaccinated in both 2016/17 and 2015/16 seasons (77.2%, 95% CI: 19.6 to 93.5) and lower in those vaccinated only in the 2016/17 season (5.4%, 95% CI:-140.9 to 62.8). The interaction term was significant (p = 0.029), with the VE for vaccinated both years being significantly different to not vaccinated in either year.

### Vaccine effectiveness against influenza B

#### In adults

The age-specific VE in adults for IIV overall and stratified by age group is shown in Table 5. In adults 18–64 years of age, the aVE point estimate was 52.1% (95% CI: -20 to 80.9), whereas in those 65 years of age and above, the aVE point estimate was not statistically significant with very wide 95% CIs.

#### In children

The VE in children 2–17 years of age by vaccine type is shown in Table 5. The aVE point estimate for LAIV4 against influenza B was 78.6% (95% CI: -86 to 97.5). There was inadequate precision to calculate VE for IIV in children. There were inadequate influenza B detections to calculate VE by prior vaccine status in either adults or children.

### Comparison of end-of-season to mid-season vaccine effectiveness

The mid-season analysis comprised 1,722 individuals: 1,452 were controls and 270 cases. Of the cases, 257 were influenza A and 13 were influenza B. Mid-season VE estimates, adjusting for the same variables as the end of season analysis, provided an aVE point estimate of 26.7% (95% CI: -10.4 to 51.3) for all ages against confirmed influenza A(H3N2) primary care consultation. All mid-season estimates were generally similar albeit with wider CIs compared with the end of season estimates.

## Discussion

Our analysis found that in a season dominated by early circulation of influenza A(H3N2), there was moderate overall VE in preventing laboratory-confirmed influenza in primary care. Although seasonal influenza vaccine was effective against influenza A(H3N2) infection in younger adults, there was no evidence of significant effectiveness of inactivated vaccine in people aged 65 or older. On the contrary, in children there was good effectiveness for LAIV4 against influenza A(H3N2) and a suggestion of effectiveness against influenza B, for which there were only limited detections. Finally, we found no evidence that prior season vaccination significantly reduced the effectiveness of influenza vaccine during the current season in adults, and rather increased effectiveness in children.

There are several strengths of this study. We used the TNCC design which is a well-established approach to measure influenza VE in the UK as in many other countries. We used our standard method to provide comparability to previous seasons’ UK VE estimates. The mid-season point estimates were similar to those at the end of the season, albeit with wider CIs, which is reassuring. There are, however, limitations to the study; in particular only relatively small numbers of children had received IIV with consequent broad CIs around VE point estimates and comparisons to those that have received LAIV4 should be made with caution, as the former also contained groups in whom the live vaccine was contraindicated such as those with severe asthma and immunosuppression.

The results of moderate VE in younger adults are consistent with the mid-season 2016/17 estimates published elsewhere in Europe and North America, all of whom experienced influenza seasons dominated by circulation of A(H3N2) viruses: The Integrated Monitoring of Vaccines in Europe (I-MOVE) network reported an overall VE point estimate of 38.0% (95% CI: 21.3 to 51.2) in a number of European countries [14]; the US CDC mid-season point estimate was 43% (95% CI: 29 to 54) [15] and the Canadian VE network reported a mid-season VE of 42% (95% CI: 18 to 59) for all ages [16].

A small number of studies have currently reported effectiveness in those aged 65 and above for the 2016/17 season, with the I-MOVE network also reporting a non-significant VE in the ≥ 65-year-olds of 23.4% (95% CI: − 15.4 to 49.1) [14], as did study teams from Sweden and Finland [17]. The observation of reduced VE in this vulnerable population may relate to the circulating virus strains, the vaccine itself and/or host factors. The available genetic characterisation data indicate that the circulating strains clustered to the same genetic lineages as the 2016/17 A(H3N2) inactivated vaccine virus strain, which was an A/Hong Kong/4801/2014 (H3N2)-like virus. Some authors have suggested that reduced VE may relate to the emergence of the genetic sub-clade 3C.2a1 [17,18]. We found a non-significantly lower VE for this sub-clade compared with the 3C.2a clade, although the antigenic characterisation data of circulating viruses remained limited, and there was no suggestion of mismatch based on the available information. Lower IIV VE in older adults is consistent with recent studies, which report that the current generation of inactivated vaccines against A(H3N2) usually result in a lower VE in those ≥ 65 years of age [19], where immune senescence may be an important factor. Indeed a recently published meta-analysis reported a pooled VE of 24% (95% CI: -6 to 45) in older adults for A(H3N2) [20]. Lower VE in adults  ≥ 65 years of age seems to be less of an issue for A(H1N1)pdm09 and influenza B, where generally little disease occurs in the older age groups, presumably due to underlying cross-protective immunity in this population [20].

We found no evidence of significant negative interference in adults from prior season’s influenza vaccine, although VE was lower among those also vaccinated the previous season. However, the antigenic distance between the A(H3N2) viruses in the current and prior season and between the current season vaccine and current epidemic strain was only small [7].

Our findings do highlight that better vaccines for older adults are required. Adjuvanted vaccines have recently been licenced in the UK, with both them and high dose vaccines available elsewhere in Europe and North America. It will of course be important to determine the effectiveness of these vaccines; early results from the US in elderly people for high dose vaccines have been encouraging [21]. In the meantime, the VE findings for the current generation of inactivated vaccines reinforce the importance of physicians considering the added value of influenza antiviral treatment and prophylaxis for those aged 65 years and older particularly in seasons dominated by A(H3N2).

This present study reports significant LAIV4 effectiveness for children 2–17 years of age against influenza A(H3N2) and a high, but non-significant effectiveness against influenza B albeit the numbers were low. These results continue to be reassuring, particularly in the light of the temporary suspension of LAIV4 in the US following the CDC finding of reduced VE [4]. The US results were at odds with those seen in several other countries that had used LAIV4 in 2015/16, including the UK [5,22]. The present study also supports results from an earlier VE study in the UK in 2014/15 that showed evidence of LAIV4 effectiveness against influenza A(H3N2) compared with IIV in children, when the circulating strain was drifted from the vaccine virus strain that season. This study also found a high, but non-significant LAIV4 effectiveness against influenza B [23]. The reasons for the findings of reduced LAIV VE in the US remain unclear, though the findings were particularly notable against A(H1N1)pdm09. Other countries noted relatively lower effectiveness of LAIV against A(H1N1)pdm09 compared with IIV in 2015/16 [24,25]. It was been suggested that this might relate to reduced replicability of the A(Bolivia) H1N1pdm09 vaccine strain in the vaccine that year [22], though there were inadequate numbers of A(H1N1)pdm09 cases this season to be able to explore this question further. Prior season vaccination has been raised as one other potential hypothesis, as the US paediatric influenza vaccine programme has been running for almost a decade compared with the more recently introduced UK programme and unlike in the UK, healthy children from 6 months to 2 years of age are offered inactivated vaccine as their priming dose. However, we found no evidence that vaccination in the prior season with LAIV4 reduced effectiveness of vaccination in the current season; a finding that matches those reported from elsewhere, such as Finland, where children could previously have received either LAIV or IIV [26]. It will, however, be critical to continue to monitor the effectiveness of LAIV in the forthcoming season, particularly with the update of the A(H1N1)pdm09 vaccine virus component for the 2017/18 season to the new A/Michigan/45/2015 (H1N1)pdm09-like virus (A/Slovenia/2903/2015) strain and the importance of determining its effectiveness against circulating strains that season [27].

In summary, we provide encouraging results for the new UK childhood influenza vaccine programme using LAIV4, albeit in a season dominated by A(H3N2) no significant effectiveness of IIV was demonstrated in adults aged 65 years and above. The level of LAIV4 effectiveness observed in 2016/17 combined with uptake in children [6] should maximise the population level benefits of the programme. These benefits are projected to provide direct protection to those vaccinated, and by reducing children’s rates of infection, to indirectly protect more vulnerable members of their families and communities, in particular older adults and those who belong to a clinical at risk group [28,29]. The findings in both children and those aged 65 years and above will need further epidemiological and virological investigation. Particularly for the former age group, the results from a season dominated by circulation of influenza A(H1N1)pdm09 viruses will be critically important to provide on-going assurance of the optimal design of the UK programme.
